# Manta ray injury affects cleaner attraction, while cleaning behaviors reflect cleaner number

**DOI:** 10.1093/beheco/arag125

**Published:** 2026-10-07

**Authors:** Yotam Barr, Avigdor Abelson

**Affiliations:** School of Zoology, Faculty of Life Sciences, Tel-Aviv University, P.O. Box 39040, Tel Aviv 6997801, Israel; School of Zoology, Faculty of Life Sciences, Tel-Aviv University, P.O. Box 39040, Tel Aviv 6997801, Israel

**Keywords:** cleaning stations, reef manta ray, cleaning ecology, interspecific interactions, remote autonomous cameras, seamount ecology

## Abstract

Cleaning mutualisms provide a useful framework for examining how cooperation and conflict are regulated, yet the extent to which client condition influences these dynamics remains unclear. We tested whether reef manta ray (*Mobula alfredi*) injury status is associated with cleaner engagement and behavioral dynamics at a cleaning station in the Philippines. Using video footage of 278 cleaning events from 96 individuals, mantas were classified as non-injured, having old injuries, or entangled (ongoing injuries). We found a distinction between the factors influencing cleaner attraction and those governing behavioral dynamics during cleaning. Injury category was significantly associated with the number of attending cleaner wrasse (*Labroides dimidiatus*); entangled mantas attracted significantly more cleaners than non-injured or previously injured individuals. By contrast, once an interaction was initiated, behavioral dynamics were more closely associated with local cleaner density than client condition. The probability of cleaner chases (reflecting intra-specific competition), the number of client jolts (aversive responses linked to conflict), and cleaning duration all increased significantly with cleaner number, whereas no additional association with injury category was detected. Because injury category itself predicted cleaner abundance, however, injury may remain indirectly relevant to these behavioral dynamics through its association with cleaner number. These findings suggest a pattern in which client condition establishes the scale of the interaction by attracting cleaners, and the resulting social environment and intra-specific competition are more closely associated with how the interaction unfolds. This highlights a fundamental distinction in mutualistic systems between cleaner attraction and the regulation of interaction dynamics.

## Teaser

Injured manta rays attract more cleaner fish, although the dynamics of cleaning interactions were more closely associated with the number of cleaners present. Using video recordings from a cleaning station, we found that ongoing injuries were associated with greater cleaner abundance, whereas cleaner competition, manta jolts, and cleaning duration all increased with cleaner number. Because injured mantas attracted more cleaners, injury may therefore also influence interaction dynamics indirectly through cleaner abundance.

## Introduction

Cleaning mutualisms in the marine system, in which cleaner fish remove parasite and dead tissue from a client fishes’ body, provide a useful framework for examining how cooperation and conflict are regulated in interspecific interactions (Bshary and Schäffer 2002). In such systems, behavioral outcomes emerge from the interplay between partner characteristics and the social context of interactions, as predicted by biological market theory (Noë and Hammerstein 1995; Bshary and Noë 2003). A key, yet underexplored, question is whether the factors that determine which partners are engaged are the same as those that govern how interactions unfold once initiated.

Reef manta rays (*Mobula alfredi*) are large, highly mobile elasmobranchs with broad spatial ranges, regularly moving between offshore pelagic environments and coastal reef systems (Jaine et al. 2012; Stevens et al. 2019). This movement exposes manta rays to a variety of ecological contexts. These include open-water environments (Couturier et al. 2018) where marine debris, such as ghost nets, is increasingly prevalent, and nearshore and reef-associated habitats where interactions with human infrastructure and fishing activity are more frequent (Dulvy et al. 2014; Savage et al. 2026). As a result, manta rays are commonly observed with non-lethal injuries, including tissue damage and long-term entanglement, which may persist for extended periods (Strike et al. 2022). Such injuries can alter the physical condition of the animal, including tissue integrity and surface characteristics, and may have consequences for health and function, although their broader ecological effects remain poorly understood (Bansemer and Bennett 2010; Womersley et al. 2021; Savage et al. 2026).

Manta rays routinely visit cleaning stations, where cleaner fishes remove ectoparasites and maintain skin condition, making these interactions a key component of their ecology (O'Shea et al. 2010; Barr and Abelson 2019). The co-occurrence of frequent injury and reliance on cleaning services raises the possibility that injury may influence how manta rays engage with cleaner fishes, and how cleaning interactions are structured.

*Labroides dimidiatus* is an important cleaner species involved in many cleaning interactions with manta rays and is the cleaner wrasse examined in the present study. The species lives in socially structured harems characterized by size- and sex-based dominance hierarchies, typically comprising a dominant male and subordinate females (Robertson 1974; Clague et al. 2011). Its behavior, including service quality and intraspecific interactions, can vary with client characteristics, such as body size and whether the client is a resident or a visitor to the reef (Grutter and Poulin 1998; Bshary and Schäffer 2002; Grutter et al. 2005), as well as in response to variation in local cleaner-to-client ratios and client availability (Triki et al. 2025). *Labroides dimidiatus* may also roam several meters from its cleaning station, suggesting that individuals associated with neighboring stations may use overlapping areas and potentially move between them (Barr and Abelson 2026a). From a behavioral perspective, it is useful to distinguish between factors that influence the number of cleaners attracted to a client and those that shape behavioral dynamics during interactions. While client condition is expected to influence the former, the latter may depend more strongly on the social environment created by the number of interacting cleaners.

Cleaning interactions are strongly influenced by client condition, as ectoparasite load, tissue damage, and infection can determine both the demand for cleaning and the rewards available to cleaner fishes (Grutter 1995; Grutter and Poulin 1998; Bshary and Grutter 2002a). Injuries in fishes and elasmobranchs are frequently associated with increased susceptibility to ectoparasites, the accumulation of necrotic tissue, and the development of secondary infections (Borucinska et al. 2002; de Macedo et al. 2023), all of which may create profitable feeding opportunities for cleaner fishes, which are known to remove material from wounds and dead peripheral tissue at cleaning stations (Foster 1985).

Cleaner fishes are known to preferentially attend clients that provide higher rewards, such as those with elevated parasite loads or compromised tissue condition, suggesting that variation in client health can directly influence cleaner attraction (ie the number of cleaners attending a client) and service allocation (Grutter 1995; Bshary and Grutter 2002a). In this context, injuries—particularly those involving ongoing tissue damage or the presence of foreign material—may create localized areas of increased resource availability, potentially attracting greater numbers of cleaners. However, despite this expectation, the role of injury in shaping cleaning interactions has received little attention. In systems such as manta-ray cleaning stations, where individuals repeatedly return to the same sites (Setyawan et al. 2018; Perryman et al. 2019), injury may influence not only the likelihood of being serviced but also the number of cleaners engaged in each interaction. However, this aspect of cleaner–client interaction has not been explicitly tested.

While such effects are expected to influence the number of cleaners attracted to a client, it remains unclear whether client condition also directly shapes the behavioral dynamics within interactions, or whether these dynamics are primarily driven by the number of cleaners involved.

Beyond determining occurrence of cleaning events, and the number of cleaners that are involved in them, cleaning interactions are also shaped by behavioral dynamics between cleaners and their clients. These interactions are not always cooperative, as cleaner fishes may exploit clients by feeding on mucus rather than parasites, generating a conflict of interest within their mutualistic relationship (Bshary and Grutter 2002b; Bshary et al. 2008). Such interactions are often associated with observable client responses, including rapid body movements or “jolts,” which are commonly interpreted as indicators of aversive or suboptimal cleaning events in studies of cleaning symbiosis (Bshary et al. 2008; Soares et al. 2008; Binning et al. 2017).

In addition to client responses, interactions among cleaners themselves can influence cleaning dynamics. When multiple cleaners attend the same client, competition among conspecifics may lead to increased intra-specific agonistic behavior, such as chasing, and may also affect the quality and duration of cleaning interactions (Bshary et al. 2008; Raihani et al. 2010, 2012). As a result, both client-derived responses (eg jolting) and cleaner–cleaner interactions (eg chases) can provide insight into the balance between cooperation and conflict during cleaning events.

This raises the possibility that the factors influencing the number of cleaners attracted to a client may differ from those shaping behavioral dynamics during interactions.

Given that injury may influence the number of cleaners attracted to a client, it may also indirectly affect these behavioral dynamics by altering the competitive local environment among cleaners. However, it remains unclear whether injury itself influences these interaction-level behaviors, or whether they are primarily driven by the number of cleaners involved.

Building on these considerations, this study examines how injury status relates to cleaning dynamics in reef manta rays (*M. alfredi*) at a cleaning station. Specifically, we investigate whether injury is associated with (i) the number of cleaner wrasse (*L. dimidiatus*) engaged in clients’ cleaning, and (ii) behavioral aspects of cleaning interactions, including client responses (number of jolts), cleaner–cleaner interactions (chase occurrence), and cleaning duration.

We hypothesize that individuals with ongoing injuries (ie mantas entangled in nets or lines) will attract more cleaners due to the increased availability of damaged tissue and associated resources, whereas those with old, healed injuries will not differ from non-injured individuals, since both lack such damaged tissue or attached foreign material. Furthermore, because higher numbers of cleaners are expected to intensify competition among cleaners, we predict that behavioral responses such as jolting and chasing may increase with cleaner number. By disentangling the effects of injury status and cleaner number, this study aims to clarify how client condition and local social dynamics jointly shape cleaning interactions.

## Materials and methods

### Study area and cleaning stations

Fieldwork was conducted at Manta Bowl (Bondot Tacdogan; 12.6597 N, 123.7550 E), a shallow reef located on the summit of a seamount north of Ticao Island, Philippines (Fig. 1). Surveys focused on a rectangular area (200 × 150 m) at ∼22 to 28 m depth, characterized by patchy soft coral assemblages and multiple cleaning stations inhabited by the blue-streak cleaner wrasse, *L. dimidiatus*.

**Figure 1:**
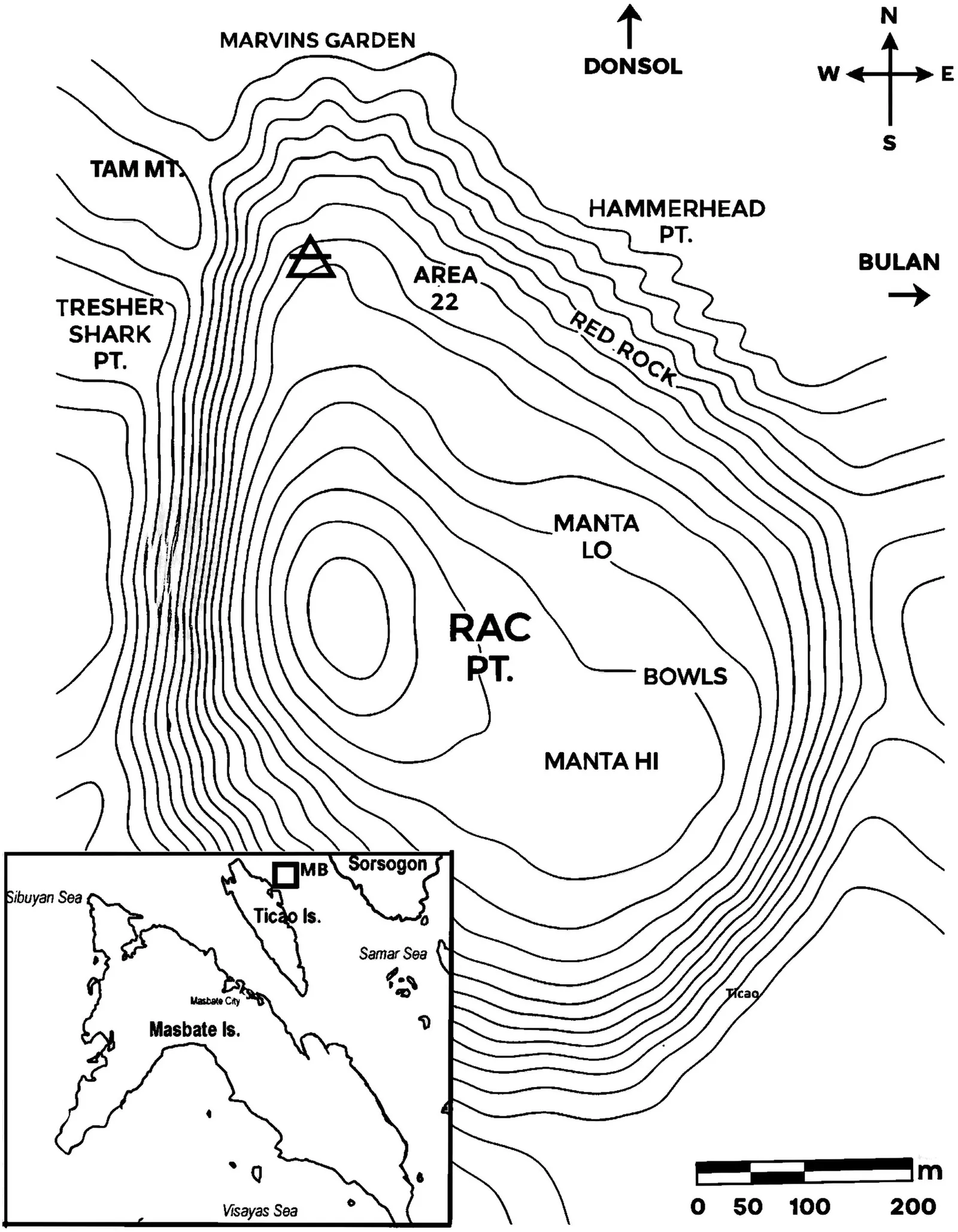
Map of the Ticao Island area (South Luzon, Philippines); the center of Manta Bowl is marked with a square and the letters MB in the inset map (12.6597° N, 123.7550° E). The main panel shows a topographic representation of the summit of the Manta Bowl seamount. The location of the Tamis Rock cleaning station is marked by a triangle (map adapted from Barr and Abelson 2025, CC BY 4.0).

Two neighboring cleaning stations, Tamis Rock and Tamis Anak (separated by ∼6 m; Fig. 2), were monitored throughout the study. Biweekly diver counts indicated a stable total of 6 cleaner wrasse on site, with 3 individuals associated with each station. Because the 2 stations were only ∼6 m apart, cleaners associated with both stations could engage with the same manta during a single cleaning event; accordingly, data from the 2 neighboring stations were pooled for analysis.

**Figure 2:**
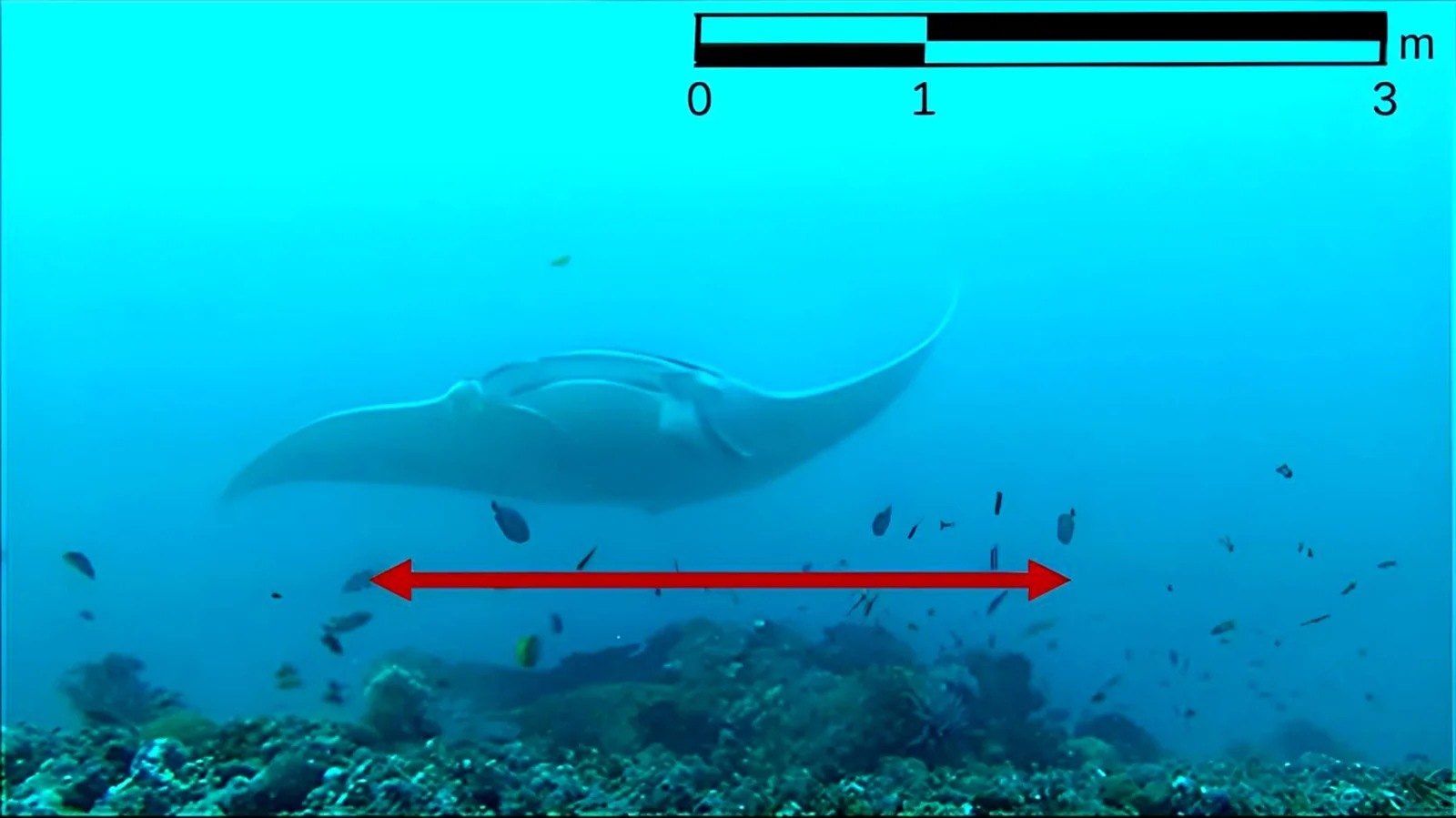
Tamis Rock cleaning station. The red line across the cleaning station represents ∼3 m. The station is characterized by a rocky structure. The formation in the background at the lower left indicates the approximate location of the Tamis Anak cleaning station (photograph taken by the author and adapted from Barr and Abelson 2025, CC BY 4.0).

#### Video surveys and event definition

Behavioral data were collected using diver-deployed autonomous cameras (Midland XTC-400) positioned to face the cleaning station area. Cameras were deployed and activated rapidly to minimize disturbance, left to record until battery depletion (3.5 to 4.3 h), and replaced to achieve ∼7 h of daily coverage. The start time of deployments alternated between 07:00 and 10:00 to broaden daytime coverage. Cameras were deployed across 52 d between July and December 2014, yielding ∼35 h of footage containing manta-ray cleaning events.

A cleaning event was defined as beginning when a manta ray approached the station and at least one cleaner wrasse-initiated inspection (approach and contact/inspection behavior). An event ended when the client departed the station and did not return. Temporary departures (eg circling following a response) were treated as part of the same event if the client returned within 1 min and cleaners remained oriented toward the client and did not switch to another client.

To avoid potential observer effects, analyses were restricted to events recorded in the absence of divers.

#### Manta identification and repeated observations

Individual manta rays were identified from video footage using natural markings (ventral patterning and distinctive external features) following Kitchen-Wheeler (2010) and assigned unique IDs (manta_id). Because individuals were observed repeatedly across the sampling period, manta_id was treated as the clustering unit in statistical analyses. Each recorded event was treated as a single observation, including repeated events involving the same individual.

### Injury categories and inclusion criteria

Each manta-ray client was assigned to 1 of 3 injury categories based on externally visible characteristics: *Non-injured*, with no visible malformations or abnormalities; *Old-injury*, with evidence of healed or partially healed tissue damage (eg deformities, amputations, or scars), with no attached foreign material; and *Entangled*, with visible foreign material (eg line or net) attached or embedded, indicating an ongoing injury.

Injury category was assigned at the individual level and remained constant across observations. This approach does not account for potential within-individual temporal changes (eg healing progression) but ensures consistent classification across repeated observations.

### Behavioral and event variables

For each event, the following variables were extracted. Number of active cleaners (count) was defined as the maximum number of cleaner wrasse simultaneously engaged with the manta during the event. A cleaner was considered active if it approached and remained near the client for ≥5 s while repeatedly inspecting or contacting it. Because individual cleaners could not always be tracked continuously, the maximum simultaneous count was used as a proxy for peak cleaning intensity within the event, as well as peak competition intensity among cleaners. Brief cleaning events in which no cleaner met the ≥5 s criterion received a value of zero for this variable. Chase occurrence (binary) was considered present when 3 criteria were met: (i) one cleaner actively followed another, (ii) at least one physical interaction occurred, and (iii) the followed individual retreated away from the client while the initiating individual returned to the client. Number of jolts (count) was defined as the number of distinct jolt events within a cleaning event. A jolt was defined as a sudden whole-body shudder and/or rapid displacement of the client while cleaners were engaged. Such responses are commonly used as indicators of client discomfort or aversive interactions in cleaning symbiosis studies. Cleaning duration (continuous) was measured in minutes from the first cleaner engagement to event termination, as defined above.

### Statistical analysis

All analyses for this observational study were conducted at the event level while accounting for repeated observations of individuals using generalized estimating equations (GEEs) implemented in IBM SPSS Statistics V.31. Manta identity (manta_id) was specified as the repeated subject effect grouping all observations from the same individual within the same cluster to account for repeated sampling of individuals. An independent working correlation structure was used due to irregular and sparse temporal spacing of observations per individual. Robust (sandwich) covariance estimates were applied for inference. GEE was selected to account for within-individual correlation while focusing on population-level effects rather than individual-specific trajectories (Liang and Zeger 1986; Zeger et al. 1988).

Separate models were constructed for each response variable to allow distributions and link functions appropriate to the nature of each response. The structure and statistical results of the models are summarized in Table 1.

**Table 1:** Summary of the GEE models used in the statistical analyses.

Response variable	Predictor	Distribution (link)	Wald χ^2^	df	*P*
Number of cleaners	Injury category	Negative binomial (log)	19.253	2	<0.001
Chase occurrence	Number of cleaners	Binomial (logit)	19.753	1	<0.001
	Injury category		1.699	2	0.428
	Injury category × number of cleaners		0.747	2	0.688
Number of jolts	Number of cleaners	Negative binomial (log)	17.006	1	<0.001
	Injury category		0.066	2	0.968
	Injury category × number of cleaners		0.398	2	0.819
Cleaning duration	Number of cleaners	Gamma (log)	25.656	1	<0.001
	Injury category		3.931	2	0.140
	Injury category × number of cleaners		4.365	2	0.113

Interaction terms between injury category and number of cleaners were included to test whether the effect of cleaner number on behavioral responses differed among injury categories. The analytical framework distinguished between (i) the effect of injury category on the number of cleaners, and (ii) the effects of cleaner number and injury category on behavioral responses during cleaning interactions.

For models where injury category was a focal factor, overall differences among categories were assessed using Type III Wald *χ*^2^ tests. Estimated marginal means (EMMEANS; on the original response scale) were obtained, and pairwise comparisons among injury categories were conducted with Bonferroni adjustment. For models including number of cleaners as a covariate, EMMEANS were computed at the mean value of the covariate.

Analyses were based on cases with complete data for all variables included in each model. Statistical significance was evaluated at α = 0.05, and results are reported with Type III Wald tests and 95% confidence intervals (CIs). Interpretation emphasizes associations rather than causation given the observational design.

For graphical presentation of the association between cleaner number and behavioral responses (Fig. 4), additional simplified GEE models were fitted with number of cleaners as the sole predictor. These models were used only to generate fitted trend lines for visualization, whereas statistical inference reported in the Results is based on the full models described above.

### Ethical statement

All observations were non-invasive and involved no capture, handling, tagging, feeding, or experimental manipulation of manta rays or cleaner fishes. Data were collected by SCUBA observations and remote video recordings, with efforts made to minimize disturbance to the animals. All dives were conducted with the support and supervision of a local licensed dive operator. The study complied with the ABS/ASAB Guidelines for the Ethical Treatment of Animals. As the study involved passive observational methods only, no specific animal ethics approval was required.

## Results

A total of 278 cleaning events from 96 individually identified reef manta rays (*M. alfredi*) were analyzed, with repeated observations per individual accounted for using GEEs. Of the 96 individually identified manta rays, 72 were non-injured, 17 had old injuries, and 7 were entangled with ongoing injuries. Of the 278 cleaning events analyzed, 175 (62.9%) involved non-injured individuals, 82 (29.5%) involved individuals with old injuries, and 21 (7.6%) involved entangled individuals with ongoing injuries. Repeated observations per individual ranged from 1 to 21 events (mean = 2.9).

### Number of cleaners

The number of cleaners engaged during cleaning events differed among injury categories (Type III Wald *χ*^2^ = 19.253, df = 2, *P* < 0.001; Fig. 3). Estimated marginal means indicated 2.68 cleaners (95% CI: 2.49 to 2.89) for non-injured individuals, 2.83 (95% CI: 2.66 to 3.01) for individuals with old injuries, and 3.71 (95% CI: 3.27 to 4.22) for entangled individuals. Bonferroni-adjusted pairwise comparisons indicated that entangled individuals were associated with higher numbers of cleaners than both non-injured individuals (mean difference = 1.03, 95% Wald CI = 0.41 to 1.66, *P* < 0.001) and individuals with old injuries (mean difference = 0.89, 95% Wald CI = 0.27 to 1.50, *P* = 0.002), whereas no difference was detected between non-injured and old-injury individuals (mean difference = 0.15, 95% Wald CI = −0.18 to 0.47, *P* = 0.811).

**Figure 3:**
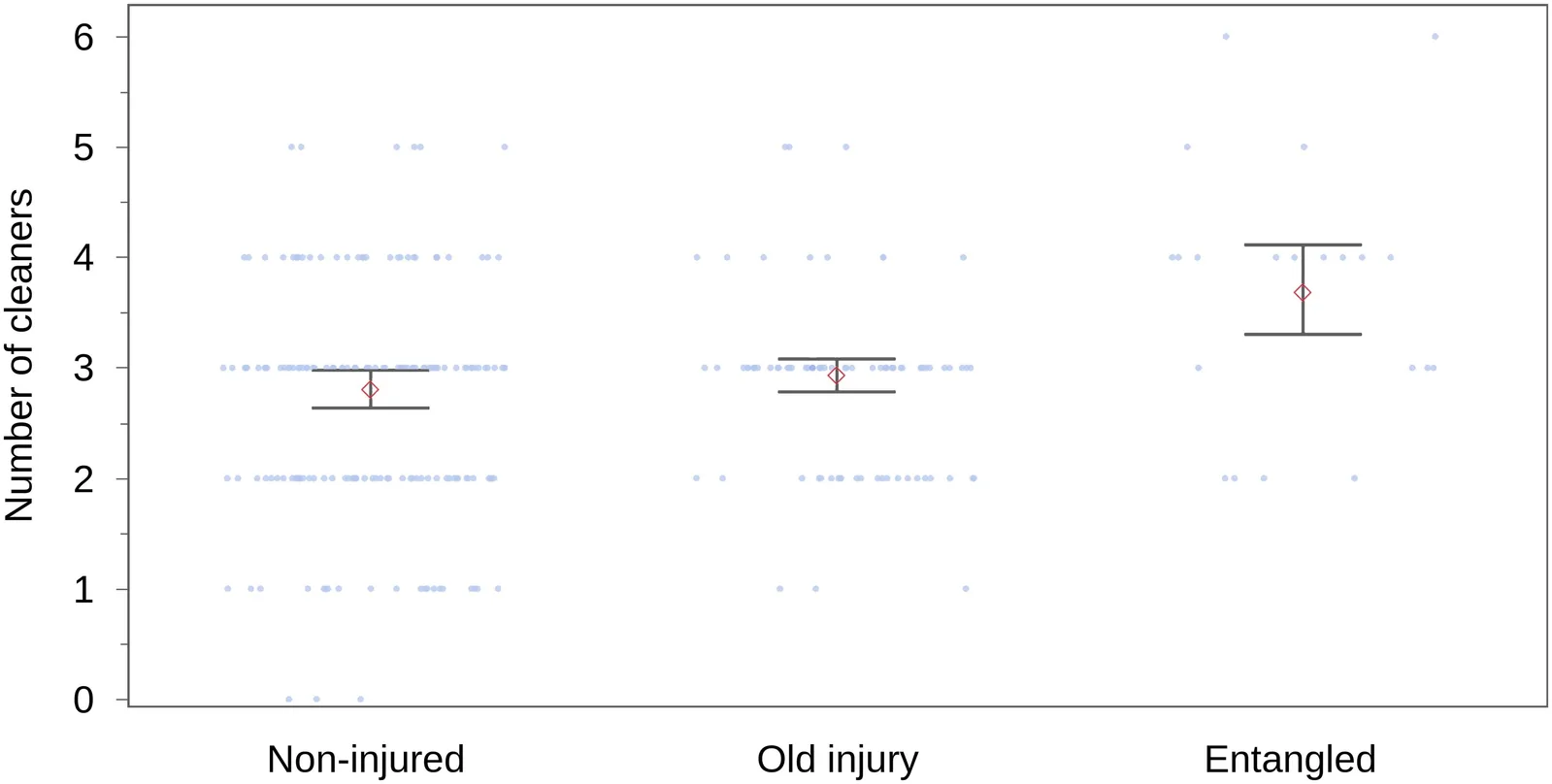
Number of cleaners per event across manta-ray injury categories. Blue points represent raw event-level observations. Red diamonds indicate model-estimated marginal means, and vertical bars show 95% CIs. Zero values represent brief cleaning events in which no cleaner met the ≥5 s active-cleaner criterion.

### Cleaner agonistic interactions (chase occurrence)

Chase occurrence was modeled using a binomial GEE with a logit link, including injury category, number of cleaners, and their interaction. The probability of chase was significantly associated with number of cleaners (Type III Wald *χ*^2^ = 19.753, df = 1, *P* < 0.001; Fig. 4a), whereas injury category was not significant (Type III Wald *χ*^2^ = 1.699, df = 2, *P* = 0.428), and there was no evidence for an interaction between injury category and cleaner number (Type III Wald *χ*^2^ = 0.747, df = 2, *P* = 0.688).

**Figure 4:**
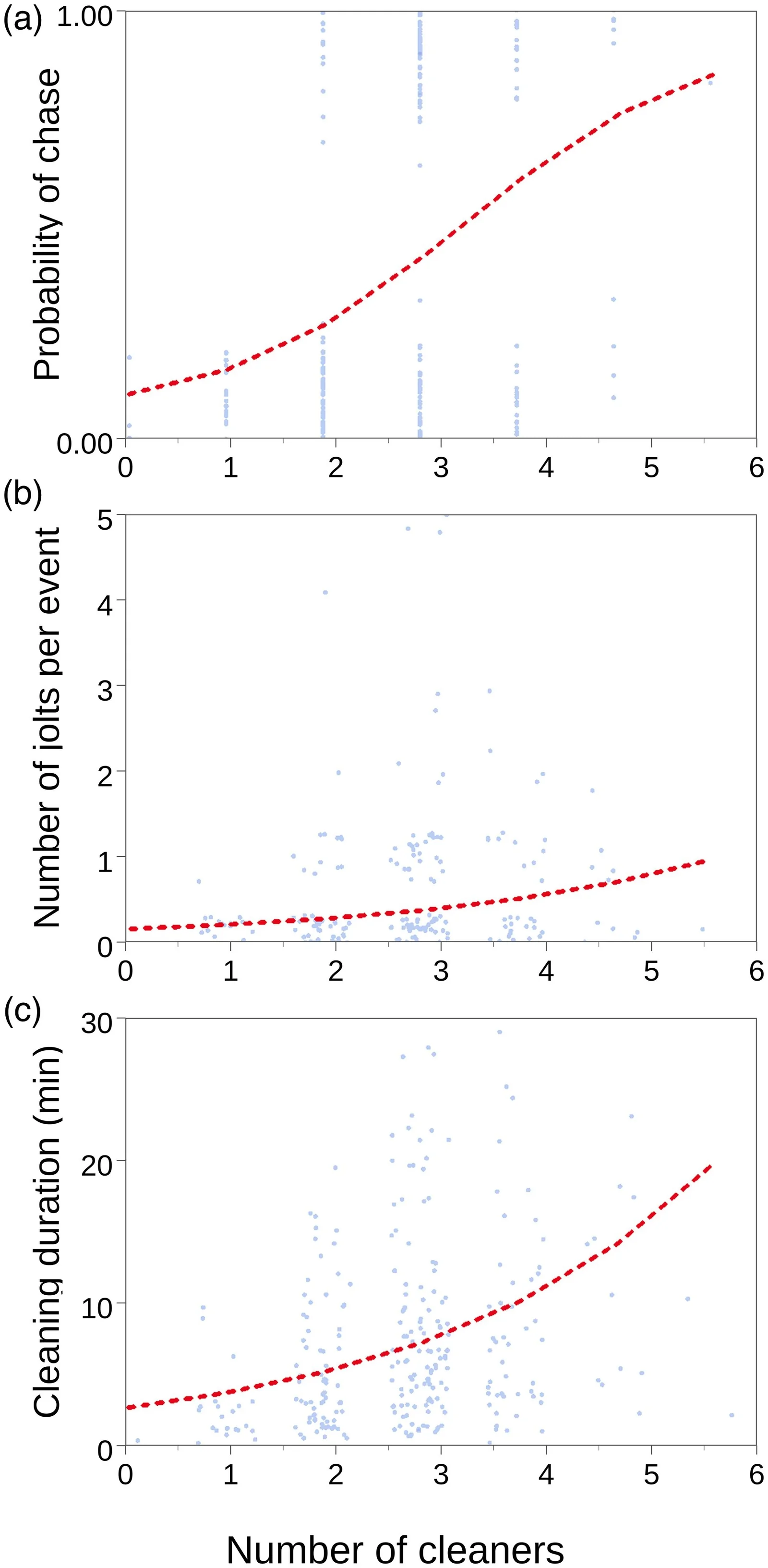
Behavioral responses as a function of cleaner number. Points represent raw event-level observations. For visualization purposes, dashed lines show predictions from simplified GEE models including number of cleaners as the sole predictor. In all panels, the x-axis indicates the number of cleaners per event. The y-axis shows predicted chase probability in (a), predicted number of jolts per event in (b), and predicted cleaning duration (min) in (c).

### Client jolts

Across the dataset, 67 of 278 cleaning events (24.1%) included at least one jolt. The number of jolts per cleaning event was modeled using a negative binomial GEE with a log link, including injury category, number of cleaners, and their interaction. The number of jolts was significantly associated with number of cleaners (Type III Wald *χ*^2^ = 17.006, df = 1, *P* < 0.001; Fig. 4b), whereas injury category was not significant (Type III Wald *χ*^2^ = 0.066, df = 2, *P* = 0.968), and there was no evidence for an interaction between injury category and cleaner number (Type III Wald *χ*^2^ = 0.398, df = 2, *P* = 0.819).

### Cleaning duration

Cleaning duration was modeled using a gamma GEE with a log link, including injury category, number of cleaners, and their interaction. Cleaning duration was significantly associated with number of cleaners (Type III Wald *χ*^2^ = 25.656, df = 1, *P* < 0.001; Fig. 4c), whereas injury category was not significant (Type III Wald *χ*^2^ = 3.931, df = 2, *P* = 0.140), and there was no evidence for an interaction between injury category and cleaner number (Type III Wald *χ*^2^ = 4.365, df = 2, *P* = 0.113).

### Overall pattern

Overall, injury category was associated with the number of cleaners engaged during cleaning events. The behavioral responses examined here, ie chase occurrence, number of jolts, and cleaning duration, all increased with number of cleaners, whereas no significant association with injury category was detected.

## Discussion

Our results demonstrate a distinction between the factors influencing cleaner attraction and those shaping behavioral dynamics during interactions. Specifically, entangled reef manta rays (*M. alfredi*) attract significantly more blue-streak cleaner wrasse (*L. dimidiatus*) than non-injured or previously injured individuals. However, once an interaction begins, behavioral responses—such as chase occurrence, number of jolts, and cleaning duration—are more closely associated with cleaner number than with the client's injury status. This pattern is consistent with the idea that client condition is associated with the initial number of attracted cleaners, while subsequent interaction dynamics depend more strongly on the local social environment created by those cleaners. It is important to note that because entangled mantas attract more cleaners, injury may also be indirectly associated with interaction dynamics through its relationship with cleaner abundance (Bshary and Grutter 2002a, 2002b; Bshary et al. 2008). These results refine current understanding of injury and mutualistic cleaning in marine megafauna by suggesting that the relative importance of injury and cleaner abundance may vary across different aspects of the interaction.

### Injury, parasite load, and cleaner attraction

Entangled manta rays were serviced by significantly more cleaners per event than non-injured or previously injured individuals, whereas no difference was found between the latter 2 groups. This pattern is consistent with evidence from reef fishes that injury increases susceptibility to ectoparasites such as gnathiid isopods (Jenkins et al. 2018), and from elasmobranch pathology showing that open wounds and ongoing trauma rapidly accumulate ectoparasites, dead tissue, and secondary infections (Borucinska et al. 2002). Similar patterns have been documented in other systems, where ectoparasitic wounds are associated with increased parasite loads and reduced host condition (Vollset et al. 2024).

In this light, entanglement wounds on manta rays may represent localized areas of increased resource availability—damaged skin, embedded fishing gear, and inflamed tissue that are more likely to harbor ectoparasites, mucus, and infection than intact or healed surfaces—thereby potentially attracting more cleaners to the area (Bshary and Grutter 2002a; Savage et al. 2026).

The lack of a difference between non-injured and old-injury clients further suggests that the ongoing nature of entanglement (foreign material actively abrading tissue) rather than past damage alone drives cleaner attraction. Manta rays visit cleaning stations to control ectoparasite infections and to keep wounds free from secondary infection, which can accelerate healing and reduce chronic stress (Foster 1985; McGregor et al. 2019). Our data are therefore consistent with the idea that entangled mantas are not only more likely to be parasitized at or near wound sites, but also that these sites are potentially offering greater foraging opportunities for cleaners, resulting in elevated numbers of *L. dimidiatus* attending entangled individuals (Borucinska et al. 2002; Bshary and Grutter 2002a). These interpretations remain indirect, as parasite load was not measured directly in the present study.

### Cleaner-wrasse behavior and number of cleaners

Once cleaners are engaged, however, our results reveal a different pattern: chase occurrence, number of jolts, and cleaning duration are all associated with the number of cleaners, whereas there is no main effect of injury category and no evidence for injury × cleaner interactions. This suggests that, for manta rays at Manta Bowl, cleaner-wrasse agonistic behavior, client responsiveness, and time-budget decisions are more closely associated with social dynamics among cleaners rather than with the client's injury status (Bshary and Grutter 2002b; Raihani et al. 2012; Binning et al. 2017). Cleaner wrasse are known to intensify competition and chasing when multiple conspecifics attend a single client, a pattern that can lead to increased biting and behaviors often interpreted as “cheating” (mucus consumption) at the cost of client irritation and jolting (Bshary and Grutter 2002b; Bshary et al. 2008). Our finding that the number of jolts and cleaning duration increase with cleaner number aligns with this expectation: more cleaners create more opportunities for competition and interactions that may increase the likelihood of client responses, without requiring cleaners to treat injured and non-injured clients differently.

The absence of a significant effect of injury on cleaning duration is also noteworthy. In some cleaning systems, longer interactions have been associated with larger or more parasitized clients (Bshary and Grutter 2002a), but our results indicate that, at this site, duration was more closely associated with cleaner number than with client injury. This may reflect a balance between cleaner competition and client tolerance: injured clients may attract more cleaners, but once engaged, the interaction appears to be shaped more by the social dynamics among cleaners and by the client's tolerance threshold than by injury status itself. In client species with access to multiple cleaning options, low-quality service can lead to abrupt departure (Bshary and Schäffer 2002), and a similar mechanism may help explain why cleaning duration in manta rays does not simply track injury condition. Together, these findings suggest that behavioral dynamics during cleaning interactions are more closely associated with cleaner number than with client condition per se.

### Interpretation within the mutualism framework

These results reinforce the distinction between the factors influencing cleaner attraction and those shaping behavioral dynamics. Similar patterns may occur more broadly across mutualistic systems. For example, pollinator attraction can be influenced by floral traits such as color and floral display (Caruso et al. 2019; Chen et al. 2020), and the recruitment and activity of ant partners can be influenced by plant-provided rewards such as extrafloral nectar (González-Teuber et al. 2012). In ant–plant mutualisms, such rewards may also influence aspects of ant behavior once partners are engaged, including defensive aggression (González-Teuber et al. 2012). In the present system, injury category was associated with cleaner abundance, with entangled mantas attracting more cleaners, whereas behavioral dynamics were more closely associated with cleaner number. From a manta-ray perspective, increased cleaner attention to entangled individuals may confer potential benefits, including enhanced wound debridement and reduced ectoparasite load. At the same time, our data caution against over-interpreting cleaner behavior as a direct index of parasite load: although wounded mantas attract more cleaners, the cleaners’ subsequent actions are shaped as much by competition and client reactions as by underlying pathology.

### Limitations and future directions

An important limitation is that parasite load was inferred from cleaner-wrasse behavior rather than quantified directly in this study. Future work could combine video-based behavioral assays with lesion mapping, mucus sampling, or observations of gnathiid and ectoparasite abundance on injured versus non-injured mantas to test the injury–parasite–cleaner link explicitly. Expanding monitoring across multiple seamounts and seasons could also reveal whether this pattern holds across manta populations and environmental conditions.

Beyond its behavioral implications, these findings may also have relevance for conservation planning in a time of increasing threats to manta rays and other large pelagic fishes, including population declines linked to overexploitation and habitat degradation (O’Malley et al. 2017; Pacoureau et al. 2021). Growing pressure on shallow coastal habitats may reduce the availability and quality of sites suitable for cleaning interactions (Berkström et al. 2014; Nunes et al. 2015). Research on cleaning behavior can therefore help identify and prioritize sites for protection, support population monitoring at known aggregation areas, and highlight the importance of conserving connected networks of sites between habitats. Protecting such connected locations may be important both for wide-ranging pelagic visitors and for the persistence and resilience of local species, including cleaner fish themselves.

Taken together, our findings show that injury category in reef manta rays strongly predicts cleaner-wrasse numbers at cleaning stations, whereas intra-interaction behavior is more closely associated with cleaner number than with client injury. This distinction may be important for understanding how cooperation and conflict are structured in cleaning mutualisms, where the number of interacting cleaners can influence behavioral outcomes and may itself be associated with characteristics of the client.

## Data Availability

Analyses reported in this article can be reproduced using the data provided by Barr and Abelson (2026b).
